# Small Bowel Lymphoma Complicated With Ischemic Colitis: A Case Report

**DOI:** 10.7759/cureus.41792

**Published:** 2023-07-12

**Authors:** Pugazhendi Inban, Carlo Kristian C Carredo, Faiza Arslan, Victor A Odoma, Isioma Okobia, Abiodun Adegbite, Shristi Sharma, Sandip KC, Tamer Zahdeh, Zubir S Rentiya

**Affiliations:** 1 Department of General Medicine, Government Medical College, Omandurar, Chennai, IND; 2 Department of General Surgery, Cebu Institute of Medicine, Cebu, PHL; 3 Department of Internal Medicine, Rawalpindi Medical University, Rawalpindi, PAK; 4 Department of Cardiology/Oncology, Indiana Iniversity (IU) Health, Bloomington, USA; 5 Department of Surgery, University of Benin Medical School, Benin City, NGA; 6 Department of Medicine, University of Ibadan, Oyo, NGA; 7 Department of Internal Medicine, University of Dhaka, Dhaka, BGD; 8 Department of Internal Medicine, Hadassah Medical Center, Jerusalem, ISR; 9 Department of Surgery, MedStar Georgetown University Hospital, Washington, USA; 10 Department of Radiation Oncology & Radiology, University of Virginia School of Medicine, Charlottesville, USA

**Keywords:** small cell lymphoma, epstein barr virus, pneumatosis coli, neutropenic enterocolitis, b cell lymphoma, ischemic colitis

## Abstract

Ischemic colitis is thought to be an injury to the colon as a result of reduced blood flow. Certain infectious diseases such as the Epstein-Barr virus can aid in the reduction of blood flow. The insult can range from inflammation and superficial injury to full-thickness necrosis. The typical regions affected are the “watershed” areas of the colon: the splenic flexure, the rectosigmoid junction, and the right colon. Because patients can present with a wide spectrum of symptoms from vague abdominal discomfort to complete abdominal catastrophe, the diagnosis of ischemic colitis is sometimes challenging to make. Patients typically present with the acute onset of crampy abdominal pain and usually pass blood mixed with stool within 24 hours. Endoscopically, ischemia is suspected in the presence of bluish hemorrhagic nodules from submucosal bleeding, cyanotic or necrotic mucosa with bleeding ulcerations, or a segmental distribution with an abrupt transition point between injured and normal mucosa. We present a case of an 80-year-old male with a history of hypertension, hyperlipidemia, and basal cell carcinoma of the scalp diagnosed with ischemic colitis associated with positive Epstein-Barr virus B cell lymphoma.

## Introduction

The most prevalent type of intestinal ischemia is ischemic colitis [1]. The condition mainly affects elderly people with underlying atherosclerosis and commonly develops spontaneously due to either thrombotic or embolic obstruction of colonic arteries or poor perfusion of the colonic wall in nonocclusive situations.

Several risk factors can contribute to the development of ischemic colitis. These factors include a lower cardiac output induced by arrhythmias, shock, congestive heart failure, and long-term cardiac bypass surgery or aortic repair. Coagulation diseases such as a deficiency of protein C, protein S, or antithrombin III, sickle cell disease, and vasculitis syndromes like systemic lupus erythematosus, polyarteritis nodosa, or thromboangiitis obliterans may induce colonic ischemia [2]. Variables that might induce colonic ischemia include digitalis, vasoconstrictors, antihypertensive medications, psychiatric drugs, nonsteroidal anti-inflammatory drugs, and antidiarrheal agents. Furthermore, antineoplastic medicines such as bevacizumab in conjunction with irinotecan and 5-fluorouracil, or docetaxel in conjunction with vinorelbine, can cause ischemic colitis [3,4]. However, R-CHOP (rituximab, cyclophosphamide, vincristine, doxorubicin, and prednisolone) treatment for non-Hodgkin's lymphoma has not been linked to an increased incidence of ischemic colitis. Colitis caused by antineoplastic therapy is typically caused by direct toxic effects of enterotoxins or extreme neutropenia. As a result, distinguishing between ischemic colitis and toxic effects on the intestinal mucosa or neutropenic enterocolitis is critical [5].

In this case report, we exemplify the very rare occurrence of intestinal perforation in an 80-year-old patient who had a diagnosis of aggressive B-cell lymphoma of the small bowel.

## Case presentation

We present a case of an 80-year-old male with a past medical history of hypertension, hyperlipidemia, and basal cell carcinoma of the scalp who presented to the emergency department with severe periumbilical pain, nausea, and vomiting. The patient reported experiencing pain for one day and had two episodes of non-bilious, non-bloody vomiting. He denied taking non-steroidal anti-inflammatory drugs, traveling, recent antibiotic use, chest pain, fever, shortness of breath, dysuria, or any bloody stools. The patient's last bowel movement was normal, and he had a meal earlier that day without any issues. On physical examination, the patient’s vital signs were stable; however, he appeared uncomfortable and had a soft, minimally distended, and moderately tender abdomen throughout.

**Figure 1:**
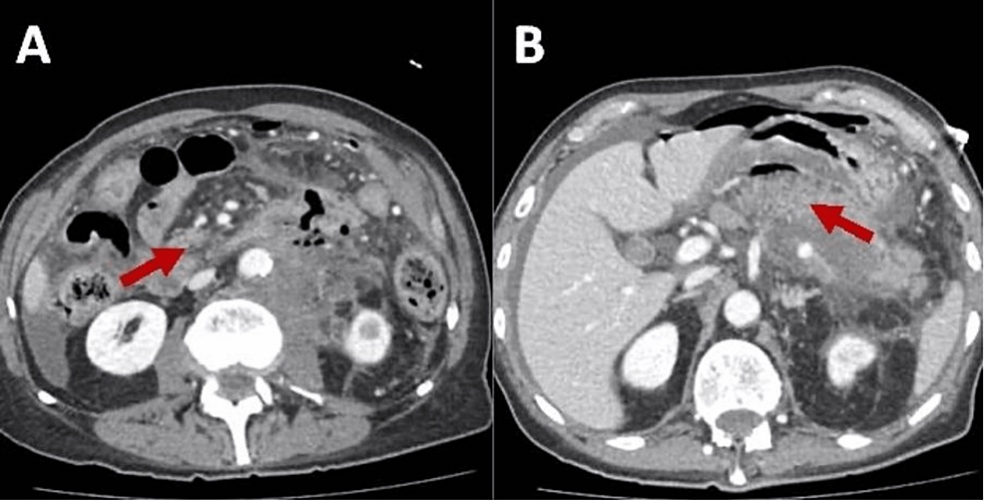
Computed tomography (CT) showing extensive complex acute pancreatitis with stable early walled-off collection and possible early necrotizing collection (A). Anterior pancreatic gastric perforation with wall thickening and second proximal jejunum perforation near peripancreatic fluid/inflammatory change (B).

A computed tomography (CT) scan of the abdomen was done, showing a swollen pancreatic body and tail with ill-defined parenchymal hypoattenuation which extended to the left retroperitoneal region, including the left renal hilum and left psoas muscle which was asymmetrically enlarged. These findings raised concern for necrotizing pancreatitis with the development of a necrotic collection, making it unlikely to be a pancreatic malignancy. Additionally, anterior to the pancreas a focal gastric perforation with associated wall thickening was found. A second perforation at the proximal jejunum, also in close proximity to the aforementioned peripancreatic fluid/inflammatory change, was found as shown in Figure 1. Mild apparent wall thickening of small and proximal large bowel loops was nonspecific but potentially reactive.

Upon admission to the hospital, the patient was found to have rising lactic acid and intraabdominal sepsis. A pre-operative diagnosis of pneumoperitoneum, gastric perforation, and jejunal perforation was made. The patient was immediately taken for exploratory laparotomy, small bowel resection with side-to-side duodenojejunostomy, gastrostomy tube placement, jejunostomy tube placement, and negative pressure wound therapy. Biopsies were sent to pathology for review.

The diagnostic results from the small bowel resection and stomach frozen section revealed a deep gastric ulcer that was associated with acute and chronic inflammation, intestinal metaplasia, and reactive changes. Additionally, the presence of *Helicobacter pylori* organisms was confirmed through a positive Steiner stain. 

Moving to the jejunum, an aggressive B-cell lymphoma was discovered in the wall. Upon closer investigation, the lymphoma appeared to be Epstein-Barr virus (EBV)-positive, specifically diffuse large B-cell lymphoma with sheets of large lymphoid cells that showed immunoblastic features. Brisk mitoses and areas of necrosis were visible as well. Immunohistochemistry confirmed that the large cells were of B-cell origin (CD20+, CD79a+, PAX5+, CD45+, and CD3-) and do not co-express CD5, CD10, CD15, CD23, CD56, CD138, BCL1, EMA, or ALK1; many of the cells were positive for CD30, BCL2, MUM1, and EBV (EBER); and BCL6 stained a minor subset of the cells. The proliferative rate, based on Ki-67, was high (up to 90-95%). There were occasional plasma cells with polytypic kappa and lambda admixed, and the findings were consistent with an aggressive diffuse large-cell lymphoma associated with EBV. There was only one reactive lymph node present. Finally, to further understand the nature of this tumor, fluorescence in situ hybridization (FISH) analyses were performed and it showed that IGH-MYC t(8;14) and IGH-BCL2 t(14;18) were negative. These findings did not indicate double-hit B-cell lymphoma. Owing to the above diagnosis, hematology & oncology were consulted to follow up with them in the outpatient setting.

**Figure 2:**
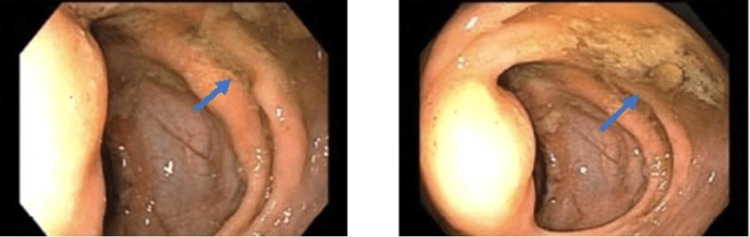
Colonoscopy images showing congested edematous mucosa with shallow ulcerations without necrosis.

On two weeks follow-up, the patient had a barium swallow study done to assess the patency of anastomosis and readiness for oral intake. It demonstrated intact anastomosis and no leak; however, the scan demonstrated concern for right colon pneumatosis. Gastroenterology (GI) was then consulted and a colonoscopy was performed, which demonstrated small shallow ulcerations, without necrosis in the proximal ascending colon with healthy surrounding mucosa and intact vascular pattern, consistent with ischemic colitis as shown in Figure 2. The patient was recommended by the GI team to continue with broad-spectrum antibiotics and supportive care.

The patient was able to ambulate with assistance and was evaluated by the physical & occupational therapy team. His pain was controlled with oral pain medications. The patient demonstrated that he was able to void independently. The patient was deemed stable and discharged.

## Discussion

Approximately 20% of extranodal lymphoma cases occur in the gastrointestinal tract [6]. In the Western world, the stomach is most commonly involved, followed by the small bowel which accounts for only 1-2% of all gastrointestinal malignancies [7]. These lymphomas originate from B- or T cells in the lymphatic system of the small intestine and primarily affect older male individuals, with the median age of diagnosis being 60 years [8]. 

Gastrointestinal B-cell lymphomas are more common than T-cell lymphomas, accounting for 80% of all gastrointestinal lymphomas, and are considered more responsive to chemotherapy with an overall better prognosis [9]. Some patients with small bowel lymphoma are asymptomatic as is the case in our patient, and others can have nonspecific symptoms such as colicky abdominal pain, bloody stools, bloating, weight loss, and changes in bowel habits [10]. These symptoms are also associated with other conditions, such as inflammatory bowel disease, celiac disease, or irritable bowel syndrome, which makes it challenging to pinpoint the cause of the symptoms.

The ileum is the most common site involved in small bowel lymphoma (up to 65% of cases), followed by the jejunum and then the duodenum [11]. The diagnosis of small bowel tumors, and lymphomas in particular, is often done at an advanced stage due to the non-specificity of symptoms and a low degree of suspicion [12]. Our patient's tumor was incidentally found in the jejunum. To help diagnose small bowel lymphoma, a range of diagnostic tests can be implemented, including endoscopy, imaging, and blood tests to check for abnormal levels of certain proteins and genetic derangements. Radiologic findings suggestive of small bowel lymphoma are usually nonspecific, making it difficult to differentiate from other benign or malignant tumors. Even if there is a high suspicion of lymphoma, these radiologic findings cannot correlate to certain pathologic subtypes, though some features seen on CT scans like multiple polyps can rule out follicular lymphoma and mucosa-associated lymphoid tissue (MALT) lymphoma subtypes [13]. 

A biopsy is the gold standard diagnostic test. It can be obtained via endoscopic techniques, most notably device-assisted enteroscopy (DAE), which includes balloon enteroscopy (single balloon or double balloon) and spiral enteroscopy [14]. Originally described by Yamamoto et al. in 2001, double balloon enteroscopy (DBE) allows diagnosis through high-resolution visualization, as well as therapeutic interventions throughout the entirety of the small bowel [15]. The introduction of capsule enteroscopy (CE) has also revolutionized the assessment of the small bowel. It is usually the first diagnostic test of choice for patients suspected of having small bowel tumors who present with occult gastrointestinal bleeding without obstructive symptoms [12]. Small bowel lymphoma can appear on CE as a polyp, mass, or ulcer, which is nonspecific and cannot be differentiated from other small bowel malignancies [16]; therefore, a biopsy is required. One systematic review and meta-analysis showed that DAE and CE have a high diagnostic concordance rate for small bowel tumors, with DAE having 89% sensitivity and 97% specificity [17]. 

Some complications can develop if small bowel lymphoma is left untreated, and they can manifest as the initial presentation. Based on many reports, these complications include bleeding, obstruction, and perforation [18-20]. Perforation of the small bowel is a serious emergency that can rapidly lead to sepsis and a high mortality rate [21]. Treatment of perforation secondary to small bowel lymphoma usually involves resection of the involved portions of the small bowel, and this can help confirm the diagnosis histologically. It should also be noted that the aforementioned complications can be triggered by the initiation of chemotherapy and therefore patients should be monitored during treatment. One of the complications that could also develop after treatment is ischemic colitis, which can occur after surgery due to venous return congestion, or as the result of direct cytotoxicity or severe neutropenia secondary to chemotherapeutic agents such as bevacizumab and 5-fluorouracil [3,5]. The patient in our case developed ischemic colitis post-surgery as seen on colonoscopy, prior to his follow-up oncology appointment or being on chemotherapy.

The R-CHOP chemotherapeutic regimen protocol, which is the standard regimen for non-Hodgkin’s lymphoma, has been not yet associated with the development of ischemic colitis [22]. However, one case report has shown that R-CHOP was the most likely cause of ischemic colitis that presented as abdominal pain in a patient with small bowel lymphoma, and the pain was relieved after switching the regimen with other chemotherapies [23]. The general treatment approach, however, for ischemic colitis is conservative, meaning supportive treatment with bowel rest, intravenous fluids, antibiotics, and supplemental oxygen. A case series of four patients with ischemic colitis after left colon resection showed no improvement on conservative treatment in one patient who needed surgical resection due to irreversible stenosis [24]. Therefore, continuous monitoring is warranted and surgical intervention for bowel resection is indicated in patients who fail to improve or have developed worsening peritonitis, perforation, or uncontrolled bleeding. Our patient who had findings of ischemic colitis on colonoscopy responded well to conservative treatment on broad-spectrum antibiotics and analgesics without further intervention.

## Conclusions

The etiology of ischemic colitis is multifactorial, and the clinical presentation is variable. The diagnosis is based on a combination of clinical suspicion, endoscopic, and histological findings. Therapy and outcome depend on the severity of the disease. This case demonstrated intestinal perforation in an older patient who had a previous diagnosis of severe B-cell lymphoma of the small intestine and later developed ischemic colitis. Early recognition and prompt surgical intervention were essential in the management of this patient.
